# Supplementation of Kiwifruit Polyphenol Extract Attenuates High Fat Diet Induced Intestinal Barrier Damage and Inflammation via Reshaping Gut Microbiome

**DOI:** 10.3389/fnut.2021.702157

**Published:** 2021-08-30

**Authors:** Minlan Yuan, Xiao Chen, Tianxia Su, Yan Zhou, Xiaohong Sun

**Affiliations:** School of Public Health, The Key Laboratory of Environmental Pollution Monitoring and Disease Control, Ministry of Education, Guizhou Medical University, Guiyang, China

**Keywords:** kiwifruit, polyphenols, high-fat diet, intestinal damage, intestinal flora

## Abstract

**Background:** Impaired intestinal integrity and barrier function is associated with various diseases, including inflammatory bowel disease and metabolic syndrome. In recent years, plant-derived polyphenols have attracted much attention on regulating intestinal barrier function. Kiwifruit was recorded as a traditional Chinese medicine which can treat gastrointestinal diseases, but the mechanism was still unclear. In this study we investigated the effects of kiwifruit polyphenol extracts (KPE) on high fat diet induced intestinal permeability and its possible mechanism.

**Results:** Dietary supplementation of KPE with 50 or 100 mg/kg bw could inhibit the increase of intestinal permeability caused by HFD and promote the expression of tight junction protein (Claudin-1, Occludin and ZO-1). From microbial diversity and RT-PCR, KPE administration reshaping gut microbiome, the relative abundance of *Lactobacillus* and *Bifidobacterium* were increased, and the relative abundance of *Clostridium* and *Desulfovibrionaceae* were decreased. The changes in microbe may influence intestinal inflammatory status. Then the expression of TLRs and cytokines were detected. KPE supplementation showed anti-inflammatory effect, the expression of IL-10 was increased and the expression of TLR-2, TLR-4, TNF-α and IL-1β were decreased. Correlation analysis indicated that the expression of tight junction protein was negative correlation with TLR-2, TLR-4, TNF-α and IL-1β expression, but positively correlated with *Bacteroidete, Bifidobacterium* and IL-10 expression; the expression of *Bacteroidete, Lactobacillusand* and *Bifidobacterium* were negative correlation with TLR4, TNF-α, and IL-1β expression.

**Conclusion:** KPE treatment relieve the intestinal damage caused by HFD, which was related to the regulation of *Bacteroidete, Lactobacillusand*, and *Bifidobacterium* expression and inhibit intestinal inflammation. KPE could be a functional component for preventing gut damage and its related disease.

**Graphical Abstract:**
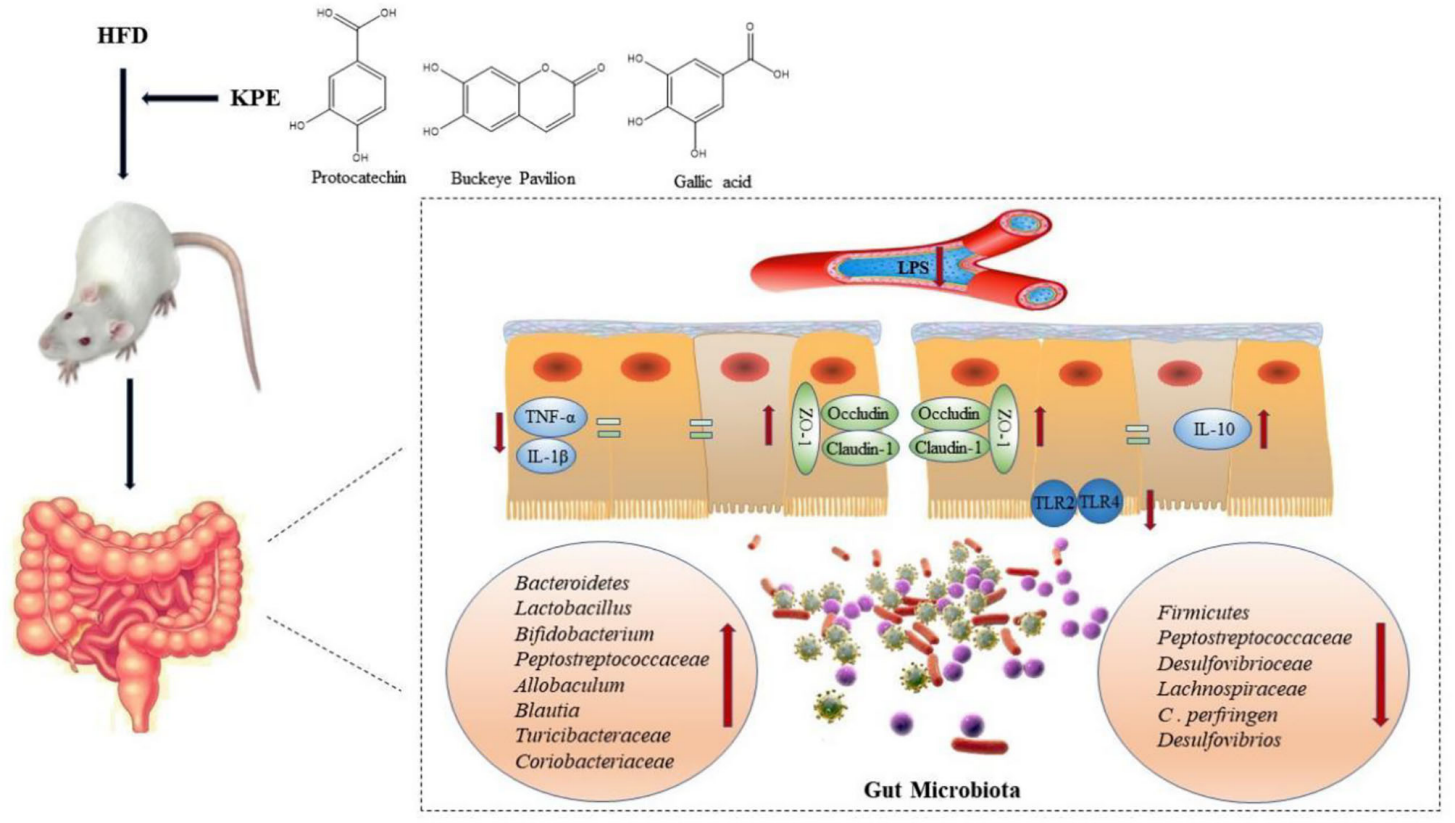
KPE relieves intestinal damage caused by high-fat diet.

## Introduction

Intestinal mucosa is the largest interface of the body, and it contacts with the external environment. In normal, it is permeable to nutrients, minerals, water and selected antigens, while it fends off bacteria, viruses and antigenic materials. Impairment in the intestinal barrier permits passage of normally excluded luminal pathogens, which elicits low grade systemic inflammation (1), and is closely related to the development of various diseases, such as inflammatory bowel disease, diabetes, obesity and non-alcoholic fatty liver (2). Thus, the integrity of intestinal barrier function is critical for maintain health.

The intestinal barrier function was controlled by tight junction. The tight junction was a complex protein structure that consistent of transmembrane proteins, including Claudin, Occludin and ZO (3). The disturbances of tight junction protein may injure intestinal barrier, and induced leaky gut. Numerous environmental factors may regulate tight junction protein and intestinal permeability, such as luminal microbiota, pro-inflammatory mediators and dietary component (3, 4). High fat diet (HFD) induced obesity increased intestinal permeability came from animal models (5–7). The increased intestinal permeability was correlated with disrupted composition of gut microbiota (8). Singh et al. (9) reported *Clostridium perfringens* interact with the intestinal epithelial cells to disrupt the tight junction protein, which were involved in the controlling intestinal barrier. The microbiota can exert a double-edged sword on intestinal permeability, while probiotics increase tight junction resistance and reduce cellular permeability. In Mangell et al.'s (10) report that supplementing with *Lactobacillus plantarum* 299v inhibits *Escherichia coli*-induced intestinal permeability. Oral administration of *Lactobacillus plantarum* increased the expression of tight junction proteins, and decreased intestinal permeability in a model of inflammatory bowel diseases (11).

Gut microbiota is also a key regulator of intestinal immune function and inflammatory response. Bacterial lipopolysaccharides (LPS) are microbe-derived ligands for Toll-like receptors (TLRs) that can trigger inflammatory responses (12). Gut microbiota is a huge reservoir of LPS, and its interactions with the gastrointestinal barrier appear to be crucial for the understanding of the complex mechanisms that maintain gut permeability. Activated TLRs initiate signal transduction pathway and triggers the expression of genes, such as inflammatory cytokines (TNF-α and IL-1β), that control immunity. Thus, TLRs serve as a molecular channel between microbiota alterations and immune homeostasis, as well as maintaining intestinal barrier function (12, 13). TLR-2/4 increased in inflammation bowel disease, and the high fat diet fed animals (14, 15). A mouse with endotoxemia would increase intestinal permeability caused by LPS, which was associated with TLR-4 dependent pathway (16). TNF-α, a kind of pro-inflammatory cytokine, impaired epithelial barrier function by tight junction disassembly (17). The effect of microbe on intestinal permeability may correlation with immunomodulate on intestinal mucous.

Recent years, much attention had been focused on the effect of plant-derived polyphenols, which alleviates intestinal damage and improves leaky gut. Naringenin, grape seed polyphenols, quercetin and curcumin improve intestinal permeability in animal model or cell culture *in vitro* (18–22). The possible mechanism involved with anti-inflammatory effect, such as inhibit NF-κB and MAPK activation (23). Kiwifruit is a native fruit in eastern Asia where it is known as Mi-hou-tao. In south of China, kiwifruits are found growing wild in dangerous narrow glens along the river Yangtze Chang. It has literatures on kiwifruit since the 1400's A.D. In ancient Chinese, kiwifruit known as Chinese herbal remedy, it used to be part of prescription to treat digestive system cancer (24). It was indicated kiwifruit maybe beneficial for digestive system health. But which was the functional component of kiwifruit? And whether it could improve intestinal injury is unknown. So, in this study, we extracted polyphenols from kiwifruit, analysis its component by HPLC-DAD, and used the high fat induced intestinal barrier injury rats. Intestinal microbiota and inflammatory mediators were detected to investigated the possible mechanism of KPE on relieve intestinal injury.

## Methods

### Extract of Kiwifruit Polyphenol

Kiwifruit polyphenol were extracted from kiwifruit peel. The kiwifruit peel was purchased from Xiuwen, Guizhou province of China. It was extracted by 60% methanol, at the rate of solid and liquid was 1:30 g/mL and 60°C, and the ultrasonic extraction time was 20 min in the power of 450 w. Then, freeze-drying was used to obtain kiwifruit polyphenol extracts (KPE).

The composition of KPE were analyzed by HPLC-PDA. Diamonsil C18 (250 × 4.6 mm, 5 μm) were used, the column temperature was 35°C, and detection wavelength was 280 nm. Methanol (A) and 0.5% acetic acid (B) were the mobile phase. Gradient elution program as follow: 0–10 min 10% A, 10–20 min 13% A, 20–27 min 18% A, 27–35 min 22% A, 35–47 min 25% A, 47–50 min 32% A, 50–58 min 35% A, 58–70 min 10 A%. The chromatogram of KPE were shown in Supplementary Results, and the composition of KPE were showed in Table 1.

**Table 1:** Chemical characterization of KPE.

	**Extract content (g/100 g dry weight)**	**Relative polyphenol content (%)**
Total polyphenols	65.40 ± 0.16	-
Gallic acid	3.14 ± 0.12	4.80 ± 0.18
Protocatechin	31.73 ± 0.19	48.52 ± 0.29
Chlorogenic acid	1.49 ± 0.04	2.28 ± 0.06
6,7-Dihydroxycoumarin	5.39 ± 1.16	8.24 ± 1.77
Catechin	2.10 ± 0.20	3.21 ± 0.31
Epicatechin	1.72 ± 0.01	2.63 ± 0.02
*p*-coumaric acid	0.59 ± 0.01	0.88 ± 0.01

### Animals, Husbandry, and Dietary Treatments

Animals: 32 male Sprague-Dawle (SD) rats (purchased from Animal Center of Guizhou Medical University) with a mean body weight (BW) of (160 ± 15.46) g were maintained in standard animal cages under specific pathogen-free conditions. Animals were housed in a controlled environment 12 h daylight cycle, lights off at 18:00 with food and water *ad libitum*. After 1 weeks of acclimation on a normal chow diet, rats were fed either a chow diet (it contained 62.3% kcal carbohydrates, 16.4% kcal proteins and 21.2% kcal fat) or a HFD diet (it contained 46.3% kcal carbohydrates, 13.3% kcal proteins and 40.3% kcal fat). Animals were randomly divided into four groups of 8 eight rats: Normal group (Normal), high fat diet group (HFD), the high fat diet-low dose kiwifruit polyphenol extract (50 mg/kg day) (HFD + LKPE) and the high fat diet with high dose kiwifruit polyphenol extract (100 mg/kg day) (HFD + HKPE). All KPE treatments were administration by intragastric. All procedures were approved by Guizhou Medical University Animal Ethics Committee.

After 9 weeks, animals were anesthetized by chloral hydrate, and then blood was taken by cardiac puncture and immediately centrifuged in order to separate serum from cells. Colon tissues were stored at −80°C. Colon tissue was fix by 10% formaldehyde over 24 h, for H&E staining.

### Measurement of Serum Endotoxin

The content of endotoxin in serum were measured by commercial kit (Xiamen Bioendo Technology Co., Ltd.), the operation was conducted by the manufacturer's instructions. The absorbance was determined by enzyme-linked immunosorbent assay and the endotoxin content was calculated according to the standard curve.

### Immunohistochemistry of Tight Junction Protein and TLRs

For paraffin-embedded colonic tissue, the sections (5 μm) were deparaffinized, rehydrated and washed in dimethylbenzene for twice, 10 min/time, then 100% ethanol, 95% ethanol, 80% ethanol, 70% ethanol, distilled water and 1% PBS for 5 min in turn. Then they were blocked in 3% H_2_O_2_ (50 μL) for 10 min, washed for three times, 5 min/time. Section were blocked with 0.01 M citrate buffer (pH = 6.0) for 10–15 min at 92–98°C, then repaired until room temperature. PBS was used for three times, 5 min/time, blocked in wet box for incubating 20 min. Then they were incubated with anti-phosphorylated Occludin antibody (1:200), ZO-1 antibody (1:100), Claudin-1 antibody (1:100), TLR-2 antibody (1:100) and TLR-4 antibody (1:100) overnight at 4°C (all antibody were purchased from Affinity Biosciences, China). After washing with PBS for 3 times, 5 min/time, cells were exposed to the secondary antibody (50 μL, at 37°C for 2 h). Then the coverslips were stained with DAB (100 μL) for 2 min and washed with PBS for 3 times. Finally, hematoxylin was used for secondary staining and blocked with neutral balsam.

### Microbial Diversity Analysis

As the described method in the previous report (25) with slightly modification. Colonic samples were pooled in equimolar and paired-end sequenced (2 × 250) on an Illumina MiSeq platform according to the standard protocols. Raw fast files were demultiplexed, quality-filtered using QIIME (version 1.9.1) with default parameters. Operational Taxonomic Units (OTUs) were clustered with 97% similarity cutoff using UPARSE (version 7.1 http://drive5.com/uparse/) and chimeric sequences were identified and removed using UCHIME. The taxonomy of each 16S rDNA gene sequence was analyzed by RDP Classifier (http://rdp.cme.msu.edu/) against the silva (SSU123) 16S rDNA database using a confidence threshold of 70%. Principle component analysis (PCA) and weighted and unweighted unifrac principle coordinate analysis (pCoA) were performed.

### Quantitative Real Time-PCR for Intestinal Tight Junction Protein, Microbial Flora, and TLR-2/4

As the described method in the previous report (25) with slightly modification. Fecal DNA was extracted using the Stool Genomic DNA Extraction Kit (Applied by TIANGEN, Beijing, China). And the intestinal tissue homogenized in Trizol reagent (Beyotime, Jiangsu, China) and total RNA was isolated, and RNA from each sample was reversely transcribed into complementary DNA by PrimeScript™ RT reagent Kit with gDNA Erase (Takara). Real time-PCR was performed using the TB Green^®^ Premix Ex Taq™ (TaKaRa) by following the manufacturer's instructions. Real time-PCR was performed using CFX96™Real-Time System (Applied Biosystems). The primers for Real time-PCR were designed and synthesized (Sangon Biotech, Shanghai, China) and the sequence information was shown in Table 2. Results were calculated using the 2^−Ct^ method.

**Table 2:** The primer sequences of Real time-PCR.

**Gene**	**Forward primer 5^′^-3^′^**	**Reverse primer 5^′^-3^′^**
Claudin-1	5′-GGACAACATCGTGACTGCTCAGG-3′	5′-TGCCAATTACCATCAAGGCTCTGG-3′
Occludin	5′-TCGTGATGTGCATCGCTGTATTCG-3′	5′-CGTAACCGTAGCCGTAACCGTAAC-3′
ZO-1	5′-CCACCTCGCACGTATCACAAGC-3′	5′-GGCAATGACACTCCTTCGTCTCTG-3′
*Bacteroidetes*	5′-AGCAGCCGCGGTAAT-3′	5′-CTAAGCATTCACCGCTA-3′
*Firmicutes*	5′-GTCAGCTCGTGTCGTGA-3′	5′-CCATTGTATACGTGTGT-3′
*Lactobacillus*	5′-CTGATGTGAAAGCCCTCG-3′	5′-GAGCCTCAGCGTCAGTTG-3′
*Bifidobacterium*	5′-GATTCTGGCTCAGGATGAACGC-3′	5′-CTGATAGGACGCGACCAT-3′
*C.perfringens*	5′-GCGTAGAGATTAGGAAGAACACCAG-3′	5′-TATTCATCGTTTACGGCGTGGACTA-3′
*Desulfovibrios*	5′-CCGTAGATATCTGGAGGAACATCAG-3′	5′-CCGTAGATATCTGGAGGAACATCAG-3′
TLR2	5′-TCTGGAGTCTGCTGTGCCCTTC-3′	5′-GGAGCCACGCCCACATCATTC-3′
TLR4	5′-TTGCTGCCAACATCATCCAGGAAG-3′	5′-CAGAGCGGCTACTCAGAAACTGC-3′
TNF-a	5′-ATGGGCTCCCTCTCATCAGTTCC-3′	5′-GCTCCTCCGCTTGGTGGTTTG-3′
IL-1β	5′-CTCACAGCAGCATCTCGACAAGAG-3′	5′-TCCACGGGCAAGACATAGGTAGC-3′
IL-10	5′-CTGCTCTTACTGGCTGGAGTGAAG-3′	5′-TGGGTCTGGCTGACTGGGAAG-3′
β-actin	5′-CCTGTGGCATCCATGAAACT-3′	5′-GAAGCACTGCGGTGCACGAT-3′
16rsDNA total bacteria	5′-ACTCCTACGGGAGGCAGCAGT-3′	5′-ATTACCGCGGCTGCTGGC-3′

### ELISA Analysis of TNF-α, IL-1β, and IL-10

The content of TNF-α, IL-1β, and IL-10 in intestine were detected using an enzyme-linked immunosorbent assay (ELISA) kit (BOSTER Biological Technology Co. ltd.) according to the manufacturer's instructions.

### Statistical Analyses

All the treatments and assays were performed in triplicate, and the results are presented as mean ± standard deviation. The test data were processed by one-way analysis of variance (ANOVA) using the SPSS version 17.0 software. A multiple comparison of data was performed by Tukey's test, which was used to compute significant differences at *P* < 0.05. Pearson test was used to analyze the correlation between intestinal barrier and microbe, and inflammation.

## Results

### Effects of KPE on Body Weight and Intestinal Morphology

As shown in Table 3, at the beginning of the experiments all animals had the similar body weight. After 9 weeks feeding with high fat diet, the body weight in HFD rats were gained 316.55 ± 9.85 g, were significantly higher (*P* < 0.05) than that in Normal group. While supplementing with KPE, the increased of body weight was effectively inhibited by KPE. The body weight and body weight gain in HFD + HKPE group were 461.00 ± 26.81 g and 253.72 ± 9.76 g, respectively, had significantly difference (*P* < 0.05) when compared to HFD rats. In HKPE, the body weight and body weight gain were equal then that in normal animals.

**Table 3:** Effects of KPE on the body weight of rats with high fat diet.

	**Initial weight/g**	**Final weight/g**	**Body weight gain/g**
Normal	207.15 ± 11.26	467.26 ± 25.00^a^	260.11 ± 8.97^a^
HFD	207.95 ± 13.42	524.50 ± 28.70^b^	316.55 ± 9.85^b^
HFD + LKPE	207.09 ± 11.94	495.18 ± 22.73^ab^	288.09 ± 10.04^ab^
HFD + HKPE	207.28 ± 10.49	461.00 ± 26.81^a^	253.72 ± 9.76^a^

*Different letters (a–d) are used to express the significant difference (P < 0.05) of the same factor among groups*.

**Figure 1:**
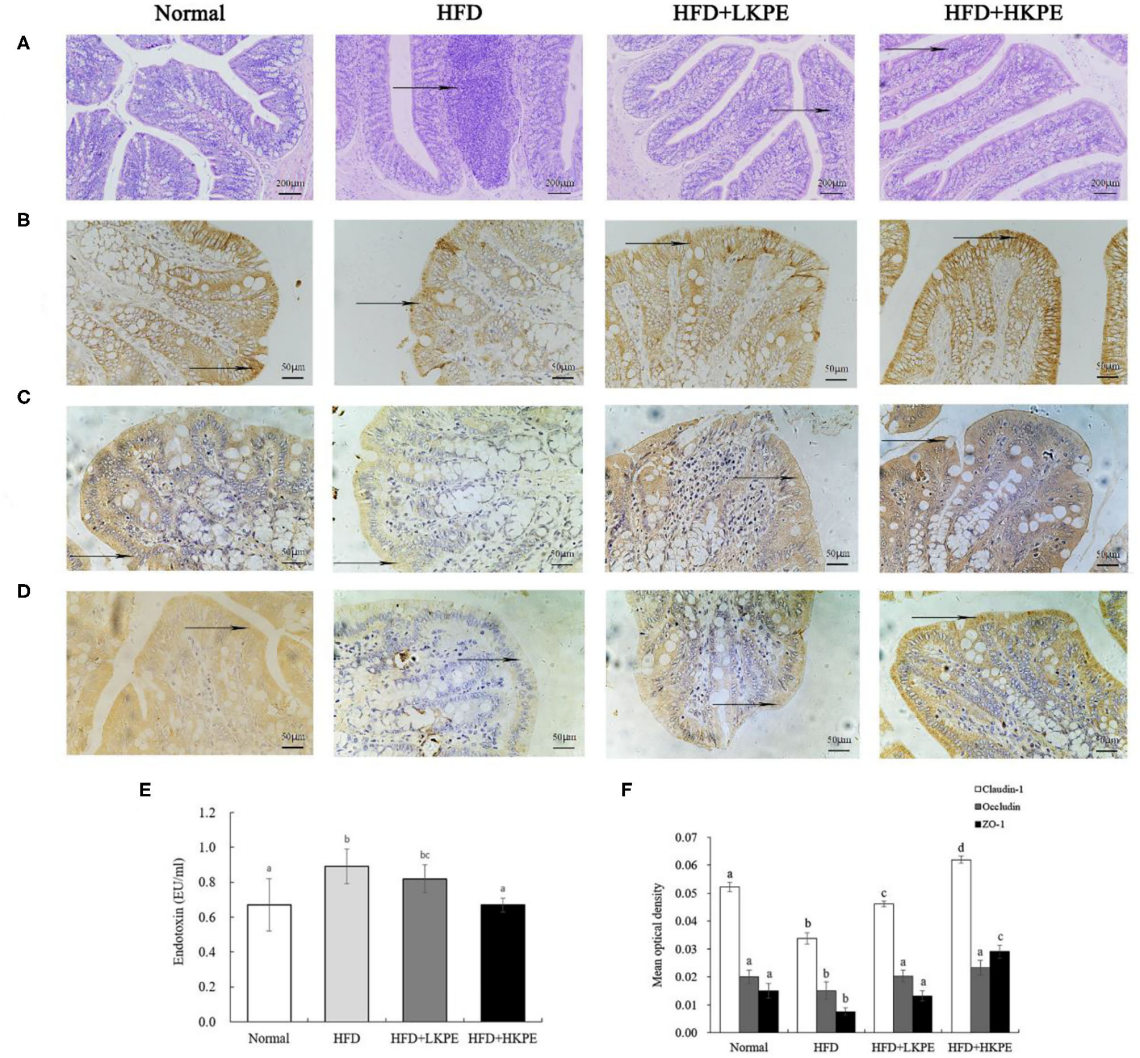
KPE supplementation alleviates high fat diet induced intestinal barrier injury. **(A)** Photomicrographs in the colon of the rat (HE × 200); **(B)** Immunohistochemical images of Claudin-1; **(C)** Immunohistochemical images of Occludin; **(D)** Immunohistochemical images of ZO-1; **(E)** The content of serum endotoxin; **(F)** Mean optical density of Claudin-1, Occludin and ZO-1. Different letters (a–d) are used to express the significant difference (*p* < 0.05) of the same factor among groups.

In previously studies, intestinal injury was observed in high fat diet feed animals. Then the intestinal morphology was determined by H&E staining. As shown in Figure 1A, the normal colonic mucosa of rats has clear cell structure and well-organized glands. Disordered glandular arrangement and abundant inflammatory cell infiltration were seen in rats induced by a high-fat diet. Dietary supplemented with KPE reduced these lesions. At HFD + LKPE can be observed the irregular arrangement of glands occasionally, while the morphology of HFD + HKPE was similar to that of the Normal (Figure 1A). These results suggested that KPE decreased the intestinal damage induced by HFD.

### Effect of KPE on Intestinal Permeability

Tight junction proteins provide a link between the transmembrane proteins and the cytoskeletal actin, thus they are important index to reveal the permeability of enterocyte. So, the expression of Clandin-1, Ocludin-1, and ZO-1 on colonic epithelium were determined by immunohistochemistry (Figure 1). From Figures 1B–D, the expression of Clandin-1, Ocludin-1 and ZO-1 had the shallowest color in HFD group, that is a negative expression. In HFD + LKPE, the color of tight junction became light brown, and it is a weakly positive expression. Moreover, HFD + HKPE had the darkest color as the dark brown, which showed Claudin-1, Ocludin-1 and ZO-1 had a strongly positive expression (Figures 1B–D).

In the result of the mean optical density value (IOD), which the IOD value of Claudin-1, ocludin-1 and ZO-1 in HFD group were significantly lower than those in cells of Normal group (*P* < 0.05). These means HFD fed rats induced the loosen of Occludin, Claudin-1 and ZO-1 in intestine. However, with supplementation of KPE may prevent the HFD induced the decreased of Claudin-1, Occluding and ZO-1 expression, especially the expression of ZO-1 (*P* < 0.05). Compared to normal samples, the IOD value of Occludin and ZO-1 at 50 mg/kg KPE bw (HFD + LKPE) supplementation had no significant difference, while at 100 mg/kg bw KPE supplementation, the expression of Claudin-1, Occludin-1 and ZO-1 were significantly increased (Figure 1F).

Serum endotoxin is an indicator of intestinal permeability. Compared with the Normal group, the serum endotoxin level in the HFD group was significantly increased (*P* < 0.05). After the supplementation of KPE, the endotoxin level was decreased compared to obesity rats. The endotoxin level in HFD + HKPE group was 0.67 EU/mL, which was 25% lower than that in the HFD group (0.89 EU/mL), showing a significant difference (*P* < 0.05) compared to HFD group (Figure 1E).

These results indicated that KPE would prevent HFD induced leaky gut and diminished of tight junction protein.

### Effects of KPE on Microorganisms

Composition of gut microbiota is an important factor contributing to the regulation of the intestinal mucus barrier function. The structural changes of gut microbiota in response to KPE were determined by 16S rDNA sequencing of colonic rat samples.

**Figure 2:**
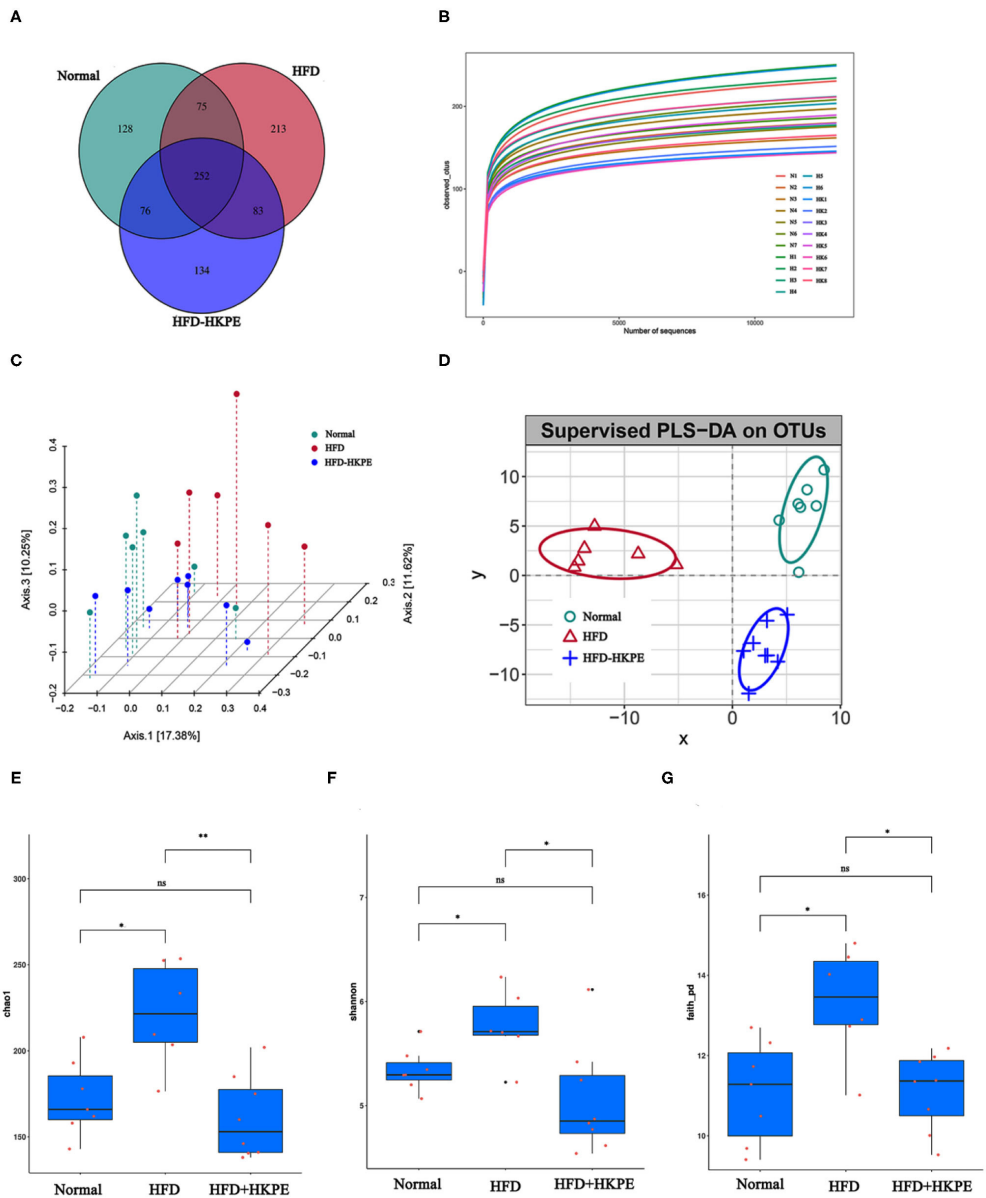
KPE supplementation alters the alpha diversity analysis and beta diversity analysis. **(A)** Venn diagram showing the unique and shared OTUs in the different groups; **(B)** Dilution curve; **(C)** Principal Co-ordinate Analysis (pCoA) based on operational taxonomic units (out) results; **(D)** Partial Least Squares Discrimination Analysis (PLS-DA) based on operational taxonomic units (out) results; **(E)** Chao1 index; **(F)** Shannon index; **(G)** Faith-pd index. *, **, ns represent *P* < 0.05, *P* < 0.01, *P* > 0.05, respectively.

To better understand the shared richness among each group, a Venn diagram displaying the overlaps between groups was developed. This analysis showed that 252 OTUs were shared among all 3 groups, with 128 OTUs found in Normal, 213 OTUs in HFD, and 134 OTUs in HFD + HKPE (Figure 2A).

With the increase of the effective sequencing depth, the rarefaction curve first increased rapidly and then leveled off, indicating that the sample sequencing data volume was reasonable, the sequencing quality was good, and the sample had certain depth and representativeness (Figure 2B). The differences in Chao1, Shannon and Faith_PD indexes between the HFD and the HFD + HKPE intervention group were statistically significant (Figures 2E–G). The results shows that HKPE can reduce the diversity of intestinal flora in rats with a high-fat diet, which is similar to the results of Wang's study (26). The results showed that the HFD increased the diversity of intestinal flora in rats, which was inhibited by HKPE intervention. We hypothesized might be due to the fact that the high-fat diet increased the number of harmful bacteria, while HKPE inhibited the survival of harmful bacteria.

The pCoA and PLS-DA results of analysis showed that HFD + HKPE and Normal were closer to each other on HFD, indicating that HKPE can normalize the intestinal flora structure of rats fed with HFD, and HKPE can regulate the intestinal flora in rats with disordered intestinal flora (Figures 2C,D).

**Figure 3:**
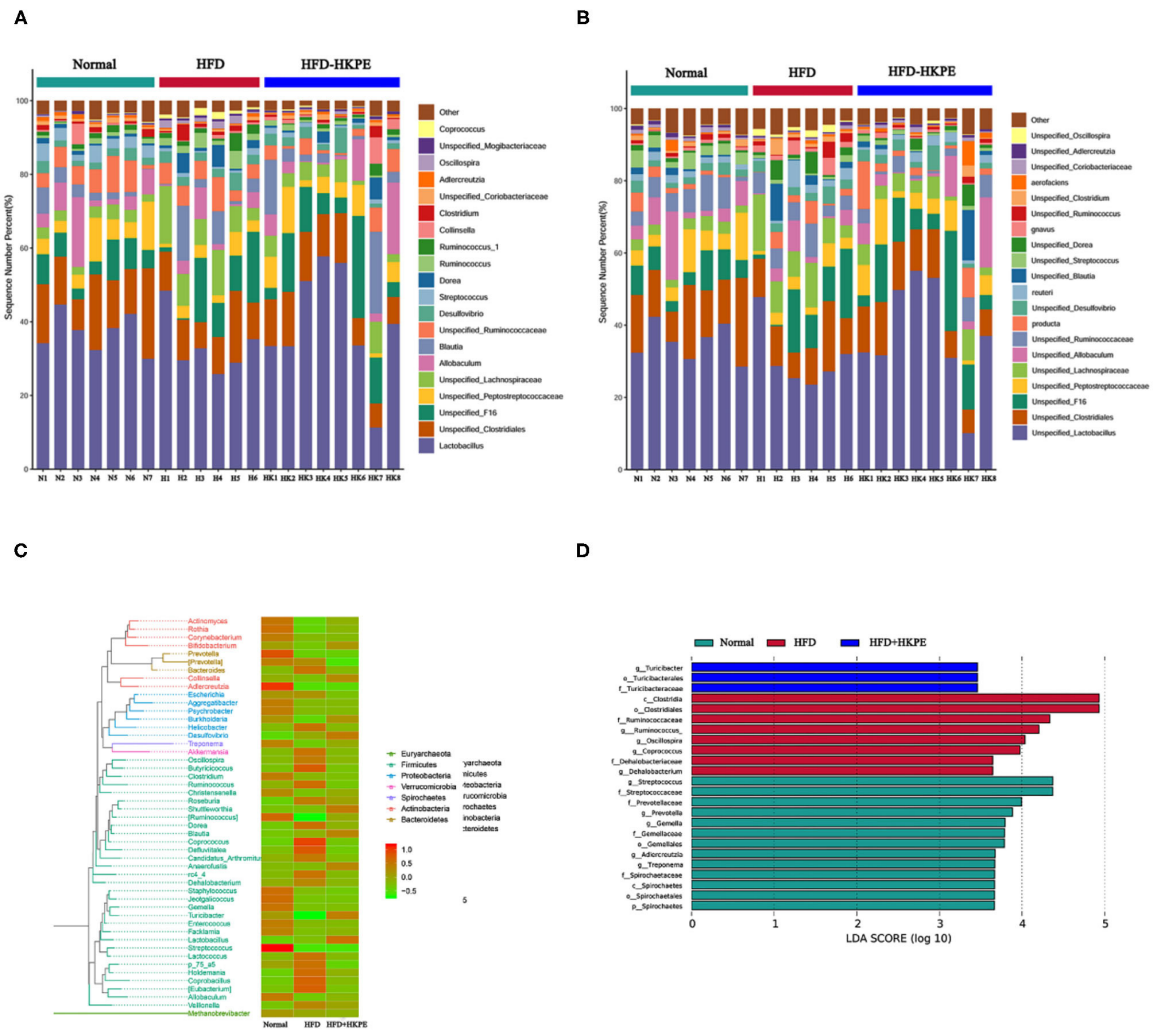
KPE supplementation alters gut microbiota. **(A)** Relative abundance of genus level; **(B)** Relative abundance of species level; **(C)** Phylogenetic_tree_heat map; **(D)** Genus lefse LDA, the heat map showing normalized relative abundance using the equation log 10.

HFD can lead to intestinal microbial disruption. Compared with Normal, the relative abundance of common probiotics, such as *Lactobacillceae, Peptostreptococcaceae* and *Coriobacteriaceae* had an obvious decrease. Moreover, in HFD, the relative abundance of common harmful bacteria, such as *Desulfovibrionaceae, Lachnospiraceae* and *Clostridiaceae* were increased. However, supplemented with HKPE can adjust the composition of intestinal flora in HFD rats, mainly by increasing the relative abundance of *Lactobacillus, Peptostreptococcaceae, Allobaculum, Blautia, Turicibacteraceae* and *Coriobacteriaceae*. And the relative abundance of *Desulfovibrionaceae, Lachnospiraceae* and *Clostridium* were reduced (Figures 3A–C). The examination of differences in microbiota compositionsusing LEfSe (Figure 3D) showed discriminant bacterial taxa among the three experimental groups: *Streptococcus, Prevotellaceae*, etc. were enriched in the Normal rats; *Clostridiales, Ruminococcaceae*, etc. were enriched in the intestinal contents of the HFD rats; and *Turicibacter*, etc. were dominant genera in the HFD + HKPE rats. It was indicated that KPE could reshaping the disrupted intestinal flora in certain extent.

**Figure 4:**
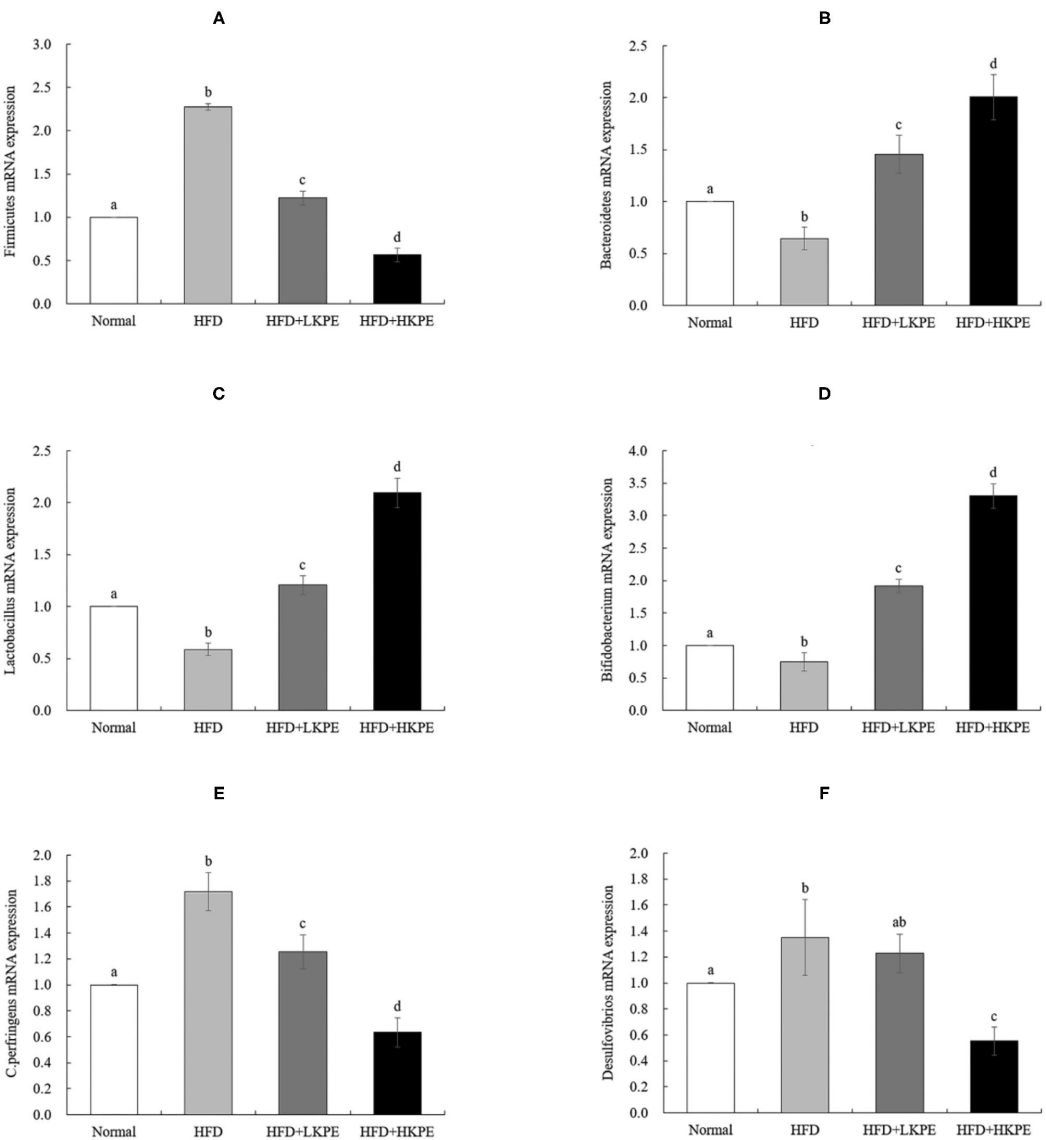
KPE supplementation stimulates the expression of *Bacteroidetes, Lactobacillus*, and *Bifidobacterium*. The mRNA expression levels of *Firmicutes* **(A)**, *Bacteroidetes* **(B)**; *Lactobacillus* **(C)**; *Bifidobacterium* **(D)**; *C. perfringens* **(E)**; *Desulfovibrios* **(F)**. Different letters (a–d) are used to express the significant difference (*P* < 0.05) of the same factor among groups.

In order to further investigated the effect of microbe, RT-PCR of some microbiota, which had shown the difference between HDF + HKPE and HDF samples determined by microbial diversity, were used. It had shown that the mRNA expression of *Firmicutes* (Figure 4A)*, Clostridium perfringens* (Figure 4E) and *Desulfovibrios* (Figure 4F) significantly increased in HFD compared with the Normal (*P* < 0.05), and the expression of *Bacteroidetes* (Figure 4B), *Lactobacillus* (Figure 4C) and *Bifidobacterium* (Figure 4D) significantly decreased (*P* < 0.05). Compared to HDF group, the expression of *Firmicutes, Clostridium perfringens* and *Desulfovibrios* in HFD + HKPE were significantly decreased, while the expression of *Bacteroidetes, Lactobacillus* and *Bifidobacterium* were significantly increased to normal level (*P* < 0.05).

### Effects of KPE on Inflammatory Mediators and TLRs Expression

**Figure 5:**
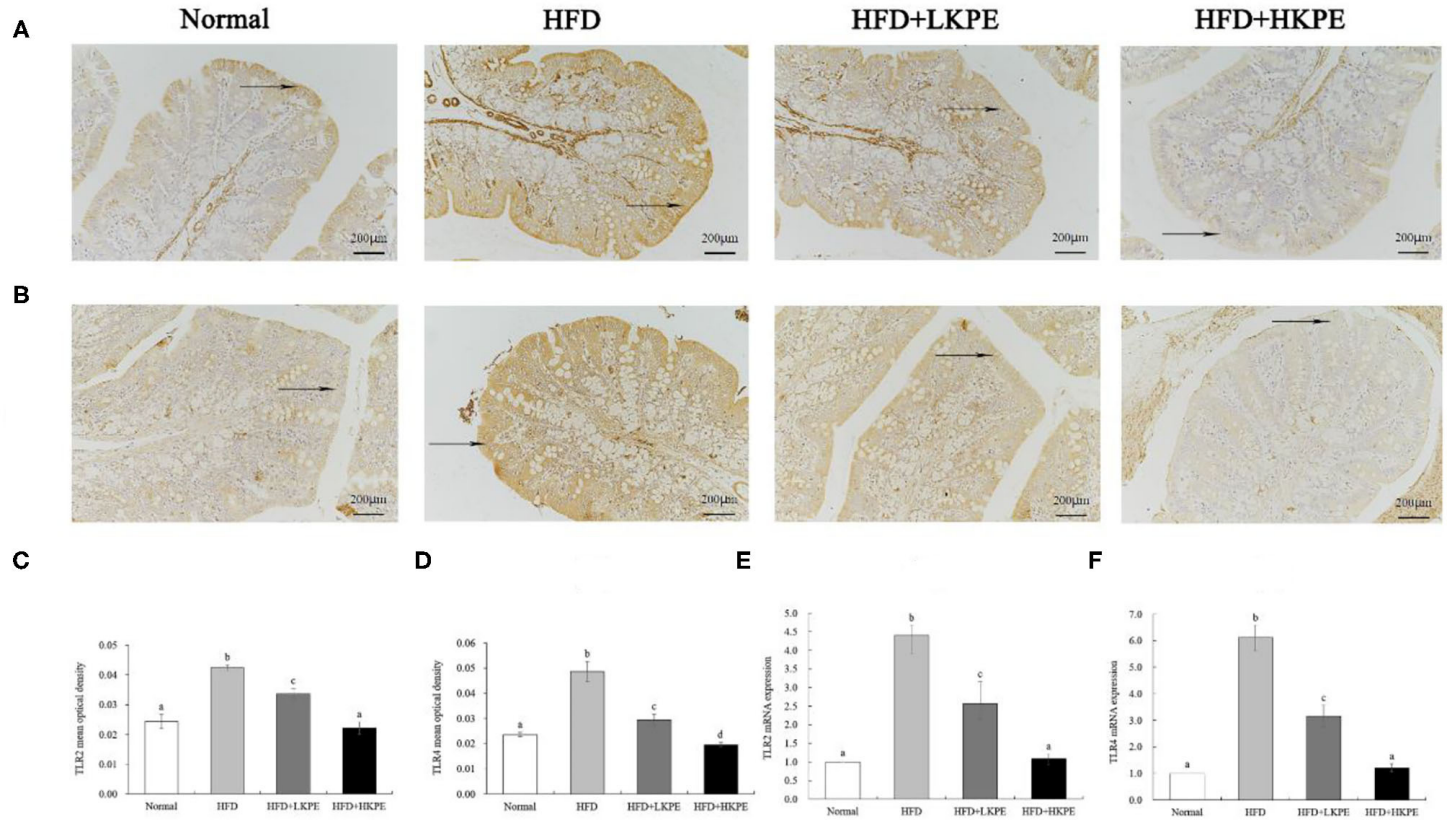
KPE supplementation decreases the expression of TLR-2 and TLR-4. **(A)** Immunohistochemical images of TLR-2; **(B)** Immunohistochemical images of TLR-4; **(C)** Mean optical density of TLR-2; **(D)** Mean optical density of TLR-4; **(E)** The mRNA expression levels of TLR-2; **(F)** The mRNA expression levels of TLR-4. Different letters (a–d) are used to express the significant difference (*P* < 0.05) of the same factor among groups.

Changes in intestinal microbes affect the level of intestinal immunity. TLR-2 and TLR-4 were the major pattern recognition receptor antigen located on the membrane of intestinal epithelial cells and it is susceptible to changes in the intestinal microflora. The expression of TLR-2/4 were determined by immunohistochemistry and RT-PCR. Form Figure 5, the color of TLR-2 (Figure 5A) and TLR-4 (Figure 5B) in HDF group samples were the dark brown, which indicts a strongly positive expression. In HFD + LKPE, the color shallowed to the light brown. The value of optical density value (IOD) was indicated of the levels of positive expression. Compared to normal animal, IOD of TLR-2 (Figure 5C) and TLR-4 (Figure 5D) had significantly increased with HFD group. After supplementation of KPE, the expression of TLR-2 and TLR-4 were decreased, even at low dose supplementation (*P* < 0.05). In HFD + HKPE group, the expression of TLR-4 was significantly decreased (*P* < 0.05) compared to normal group.

The results of RT-PCR showed similar results compared to that the data of immunohistochemistry shown. HFD fed rats showed a significantly upregulated of the expression of TLR-2 (Figure 5E) and TLR-4 (Figure 5F) compared to the normal rats (*P* < 0.05). In HFD + HKPE group, the level of TLR-2 and TLR-4 were downregulated significantly compared with the HFD group (*P* < 0.05), and had no significantly difference to normal animals.

**Figure 6:**
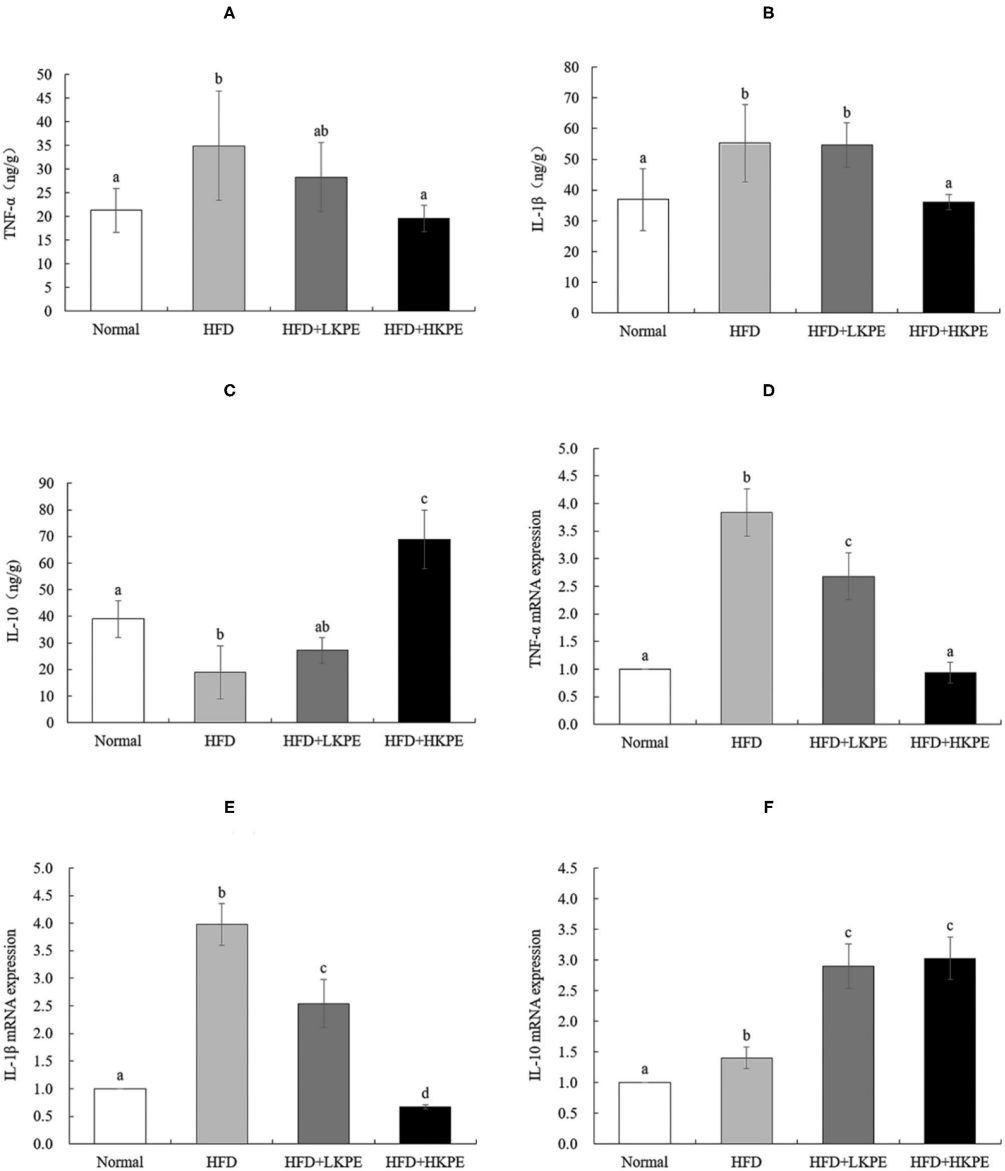
KPE supplementation regulates the expression of intestinal inflammation cytokines. **(A)** The concentration of TNF-α; **(B)** The concentration of IL-1β; **(C)** The concentration of IL-10; **(D)** The mRNA expression levels of TNF-α; **(E)** The mRNA expression levels of IL-1β; **(F)** The mRNA expression levels of IL-10. Different letters (a–d) are used to express the significant difference (*p* < 0.05) of the same factor among groups.

Inflammatory cytokines were secreted by activating TLR-2/TLR-4. Then inflammatory and anti-inflammatory cytokines were determined by ELISA. TNF-α and IL-1β are common pro-inflammatory cytokines, and IL-10 is a cytokine that inhibits inflammation. Compared with the normal rats, the level of TNF-α (Figure 6A) and IL-1β (Figure 6B) had a significant increase in HFD group (*P* < 0.05). After supplementation KPE at a dose of 50 mg/kg bw, the content of TNF-α and IL-1β had slightly decreased, but had no significantly difference compared to HDF group (*P* > 0.05), while at high dose supplementation (100 mg/kg bw) the content of TNF-α and IL-1β was decreased, had significantly difference compared to obesity rats (*P* < 0.05). IL-10 was the important anti-inflammatory cytokine, the content of IL-10 was 38.962 ± 6.886 in normal samples, while decreased to 18.902 ± 9.942 in obesity samples, had significant difference between normal group. While supplemented with KPE at dose of 100 mg/kg, the content of IL-10 was up to 68.973 ± 10.966, significantly higher than normal and HFD group (Figure 6C).

The results of RT-PCR showed similar results compared to that the data of ELISA shown. HFD fed rats showed a significantly upregulated of the expression of TNF-α (Figure 6D) and IL-1β (Figure 6E) compared to the normal rats (*P* < 0.05). In HFD + HKPE group, the expression of TNF-α and IL-1β were downregulated significantly compared with the HFD group (*P* < 0.05), while IL-10 (Figure 6F) was upregulated significantly (*P* < 0.05).

As shown above, KPE intervention can reduce the expression of TLR-2 and TLR-4 by HFD, and the effect becomes more obvious with the increase of KPE dose.

### Correlation Analysis of Intestinal Barrier With Microbe and Inflammation

KPE treatment could inhibit HFD induced leaky gut, modulate microbe and decrease inflammation status in intestine. Then, correlation between intestinal barrier function (the mRNA expression of the tight junction protein) and microbe, inflammation mediators were introduced. As shown in Table 4, *Bacteroidete*, and *Bifidobacterium* showed a positive correlation with Claudin-1 and ZO-1 expression (*P* < 0.05). No significantly correlation found between *Firmicutes, Lactobacillus, C. perfringens, Desulfovibrios* and tight junction protein expression.

**Table 4:** Correlation analysis of intestinal barrier and intestinal microbes.

**variable**	**Claudin-1 mRNA**	**OccludinmRNA**	**ZO-1 mRNA**	***Firmicutes***	***Bacteroidete***	***Lactobacillus***	***Bifidobacterium***	***C. perfringens***	***Desulfovibrios***
Claudin-1 mRNA	1								
Occludin mRNA	0.392	1							
ZO-1 mRNA	0.945^**^	0.441	1						
*Firmicutes*	−0.339	0.162	−0.310	1					
*Bacteroidete*	0.576^**^	0.195	0.626^**^	−0.662^**^	1				
*Lactobacillus*	0.450	0.241	0.442	−0.348	0.415^*^	1			
*Bifidobacterium*	0.559^*^	0.217	0.551^*^	−0.423^*^	0.494^*^	0.847^**^	1		
*C.perfringens*	−0.246	−0.278	−0.358	0.449^*^	−0.579^**^	−0.492^*^	−0.458^*^	1	
*Desulfovibrios*	−0.207	−0.082	−0.180	0.345	−0.438^*^	−0.281	−0.207	0.383	1

* *Significantly correlated at the 0.05 level (two-sided)*.

** *Significantly correlated at the 0.01 level (two-sided)*.

From Table 5, it indicated that mRNA expression of TLR-2, TLR-4, TNF-α, and IL-1β were negative correlation with Claudin-1 and ZO-1 expression (*P* < 0.01). TNF-α were significantly negative correlation with Occludin expression (*P* < 0.05), while IL-10 has a significant positive correlation with Claudin-1 (*P* < 0.01), and ZO-1 (*P* < 0.05). It was suggested that the intestinal barrier function is negative correlation with TLR-2, TLR-4, TNF-α, IL-1β expression, but positive correlation with IL-10.

**Table 5:** Correlation analysis of intestinal barrier and intestinal inflammation.

**Variable**	**Claudin-1 mRNA**	**Occludin mRNA**	**ZO-1 mRNA**	**TLR-2 mRNA**	**TLR-4 mRNA**	**TNF-α mRNA**	**IL-1β mRNA**	**IL-10 mRNA**
Claudin-1 mRNA	1							
Occludin mRNA	0.392	1						
ZO-1 mRNA	0.945^**^	0.441	1					
TLR-2 mRNA	−0.792^**^	−0.321	−0.773^**^	1				
TLR-4 mRNA	−0.722^**^	−0.328	−0.665^**^	0.939^**^	1			
TNF-α mRNA	−0.689^**^	−0.550^*^	−0.621^**^	0.747^**^	0.812^**^	1		
IL-1β mRNA	−0.774^**^	−0.328	−0.772^**^	0.976^**^	0.911^**^	0.809^**^	1	
IL-10 mRNA	0.729^**^	0.130	0.546^*^	−0.604^*^	−0.600^*^	−0.436	−0.518^*^	1

* *Significantly correlated at the 0.05 level (two-sided)*.

** *Significantly correlated at the 0.01 level (two-sided)*.

From the data from Tables 4, 5, it showed that intestinal bacteria modulate intestinal inflammation, and it indicated that inflammatory mediators play a more important role on the expression of tight junction protein rather than microbe. Then, the correlation analysis between intestinal inflammation and intestinal microbes was shown in Table 6. The expression of TLR-2 and TNF-α were significantly negatively correlated with *Bacteroidete, Lactobacillus*, and *Bifidobacterium*, and TLR-4 expression was significantly negatively correlation with *Lactobacillus*. Moreover, IL-1β was negatively correlated with *Bacteroidete* and *Lactobacillus* expression. This suggests that *Bacteroidete, Lactobacillus*, and *Bifidobacterium* were the main microbe which could reduce intestinal inflammation.

**Table 6:** Correlation analysis of intestinal inflammation and intestinal microbes.

**Variable**	**TLR-2 mRNA**	**TLR-4 mRNA**	**TNF-α mRNA**	**IL-1β mRNA**	**IL-10 mRNA**	***Firmicutes***	***Bacteroidete***	***Lactobacillus***	***Bifidobacterium***	***C. perfringens***	***Desulfovibrios***
TLR-2 mRNA	1										
TLR-4 mRNA	0.939^**^	1									
TNF-α mRNA	0.747^**^	0.812^**^	1								
IL-1β mRNA	0.976^**^	0.911^**^	0.809^**^	1							
IL-10 mRNA	−0.604^*^	−0.600^*^	−0.436	−0.518^*^	1						
*Firmicutes*	0.354	0.433	0.291	0.441	−0.268	1					
*Bacteroidete*	−0.586^*^	−0.478	−0.433^*^	−0.693^**^	0.318	−0.662^**^	1				
*Lactobacillus*	−0.589^*^	−0.562^*^	−0.563^**^	−0.498^*^	0.180	−0.348	0.415^*^	1			
*Bifidobacterium*	−0.523^*^	−0.444	−0.549^**^	−0.407	0.205	−0.423^*^	0.494^*^	0.847^**^	1		
*C.perfringens*	0.193	0.085	0.358	0.436	0.127	0.449^*^	−0.579^**^	−0.492^*^	−0.458^*^	1	
*Desulfovibrios*	0.182	0.186	0.196	0.288	−0.143	0.345	−0.438^*^	−0.281	−0.207	0.383	1

* *Significantly correlated at the 0.05 level (two-sided)*.

** *Significantly correlated at the 0.01 level (two-sided)*.

## Discussion

The intestinal tract is the largest digestive organ in the human body. The normal intestinal tract carries on the metabolism and circulation of nutrients, electrolytes and water, and also prevents harmful substances in the intestinal lumen from invading the body which is an important link in the body's defense, and play a core role in human health (2, 3). Recent studies have found that a high-fat diet causes gut microbiota imbalance and colon tissue damage, resulting in increased intestinal permeability, which is one of the main reasons for the existence of constantly circulating low-grade inflammation (27). In this study we investigated the effects of KPE and HFD on intestinal barrier in male SD rats. The result demonstrated dietary supplemented with KPE could decrease the body weight and increase the expression of tight junction protein and alleviated high fat induced leaky gut, this effect may correlate with reshaping intestinal microbiota and anti-inflammation.

Endotoxin is the main component of the cell wall of gram-negative bacteria, and the main component is lipopolysaccharide. When bacterial death, autolysis and other conditions from the cell release into the blood, can have many pathogenic effects on the body (28). Increased intestinal permeability is accompanied by increased endotoxin in the blood, thus serum endotoxin is considered as one of the indicators of intestinal mucosal permeability (29). In our study, the content of endotoxin in serum were decreased after supplemented with KPE at dose of 50 mg/kg bw, while at the dose of 100 mg/kg bw, it had significant difference compared to obesity rats. The integrity of the intestinal barrier is attributed to the expression of junction proteins. And the tight junctions are mainly composed of transmembrane protein, such as Occludin and Claudin, which form the tight junction complex with the junction complex proteins, ZO-1 can be cited as an example (30). The expressions of tight junction complex were positively correlated to barrier function. Polyphenol-rich propolis extract increased the mRNA expression of ZO-1, Claudin-1 and Occludin, and improved tight junction structure in Caco-2 cells which show that Claudin-1, Occludin and ZO-1 are positively correlated to barrier function (31, 32). In our study, supplemented with KPE promote the expression of ZO-1, Claudin-1, and Occludin. It was indicated that KPE could alleviate high fat diet induced intestinal permeability.

The disordered intestinal microbiota is related to the increased of intestinal permeability. In gut there were more than more than 10^14^ microbes colonized, the bacterial concentration ranging from 10 to 10^3^ cell per gram in the upper intestine to 10^11^-10^12^ per gram in the colon (33). In terms of bacterial phyla in intestine, the majority phyla were *Firmicutes* and *Bacteroidetes*, others were *Actinobacteria, Proteobacteria, Fusobacteria, Verrucomicrbia*, and *Cyanobacteria* (33). The intestinal microbes had shown both positive and negative effect of gut permeability. *Escherichia coli*, and *Clostridium perfringens* can secrete enterotoxin and other harmful substances, which is not conducive to the health of the body, and slow down the intestinal movement, increase the secondary absorption of harmful substances, and thereby damage the mechanical barrier of intestinal mucosa (9, 34). While probiotics, such as *Lactobacillus* and *Bifidobacterium*, can promote the growth of intestinal epithelial cells, improve the adhesion function of intestinal mucosa, protect the structural and functional integrity of intestinal epithelial cells, and thus protect the intestinal mucosal barrier from pathogenic bacterial (11, 35).

In this study we determined the effect of KPE on intestinal microbe both by microbial diversity and RT-PCR. The results of microbial diversity had shown that HFD can reduce the relative abundance *Lactobacillceae, Peptostreptococcaceae*, and *Coriobacteriaceae*, and increase the relative abundance of *Desulfovibrionaceae, Lachnospiraceae*, and *Clostridiaceae*. While supplementation of KPE at dose of 100 mg/kg bw to high fat diet fed rat, the abundance of *Lactobacillus, Peptostreptococcaceae, Allobaculum, Blautia, Turicibacteraceae*, and *Coriobacteriaceae* were increased, the relative abundance of *Desulfovibrionaceae, Lachnospiraceae*, and *Clostridiaceae* were decreased. From RT-PCR, the expression of *Firmicutes, Clostridium perfringens, and Desulfovibrios* in HKPE + HFD group were decreased, while the expression of *Bacteroidetes, Lactobacillus*, and *Bifidobacterium* were increased. In previously study had shown *Clostridium perfringens* had disadvantageous effect on intestinal barrier (9), while *Bacteroidetes, Lactobacillus*, and *Bifidobacterium* had advantageous effect on intestinal barrier (11, 36, 37). So, we indicate the improvement of KPE on intestinal permeability may contribute to regulation of microbe, especial the increased abundance of *Bacteroidetes, Lactobacillus* and *Bifidobacterium* and decrease of *Clostridium perfringens*.

Due to the close relationship between microbe and immunity in intestine, then the expression of TLR-2/4 and cytokines were determined. TLRs are expressed on various immune-related cells, and any stimuli of tissue injury can induce activation of immune-related cells (12), the interactions between gut microbe and TLRs on intestinal epithelial cells and immune cells help to maintain the homeostasis of intestinal immunity and intestinal integrity (38). The polysaccharide from *Bacteroides fragilis* is a ligand of TLR-2 orchestrates anti-inflammatory immune responses then ameliorate diseases mediated by the immune system (37). Dheer et al. (39) reported increased epithelial TLR-4 signaling was associated with an impaired epithelial barrier, and altered epithelial cell differentiation, as well as shapes microbiota, higher levels of *Fusobacteria* and *Proteobacteria* and lower levels of *Firmicutes* in the colonic mucosa. The increased expression of TLRs and cytokine produce the leaky gut, and this phenomenon should be improved by probiotics. Peña and Versalovic (40) reported that *L. rhamnosus GG* specifically inhibits TNF-α production. Our work showed HFD induced intestinal inflammatory, such as infiltrating of lymphocyte in colon (Figure 1A), promote TLR-2 and TLR-4, TNF-α, and IL-1β expression. Supplemented with KPE the intestinal inflammation was improved. Increased intestinal probiotics bacteria components should be useful for preventing TLR or cytokine mediated intestinal damage. The effect of KPE on down regulation the expression of TLR-2/TLR-4, TNF-α, and IL-1β may contribute to microbiota modulation effect, such as upregulate *Lactobacillus* and *Bifidobacterium* abundance.

Impaired intestinal mucosal barrier and increased intestinal permeability are associated not only with intestinal damage, but also with metabolic diseases such as obesity and diabetes. Increased in intaking of nature product from vegetables and fruit, such as polyphenols is a possible way to improvement intestinal permeability, then prevent intestinal damage associated disease and promote health. Kiwifruit was regarded as a medicine to deal with digestive disease, even cancer, in ancient China. In our study, we extracted polyphenols from kiwifruit peel, and identified protocatechin was the majority ingredient. Dietary supplement KPE has a protective effect on the increase of intestinal permeability caused by high-fat diet, and its mechanism is related with reshaping intestinal microbes and inhibiting intestinal inflammation by KPE. KPE maybe stimulate *Bacteroidete, Lactobacillus*, and *Bifidobacterium* expression, and then down-regulate the expression of inflammatory mediators, thus improving tight junction protein expression. KPE can be used as a functional factor to relieve the damage of intestinal barrier.

## Data Availability Statement

The datasets presented in this study can be found in online repositories. The names of the repository/repositories and accession number(s) can be found below: NCBI SRA; PRJNA749131.

## Ethics Statement

The animal study was reviewed and approved by Ethics Committee of Guizhou Medical University.

## Author Contributions

YZ designed the research. MY and TS executed the experiments and analyzed the data. YZ and XS reviewed and edited this manuscript. XC wrote the manuscript. All authors have read and agreed to the published version of the manuscript.

## Conflict of Interest

The authors declare that the research was conducted in the absence of any commercial or financial relationships that could be construed as a potential conflict of interest.

## Publisher's Note

All claims expressed in this article are solely those of the authors and do not necessarily represent those of their affiliated organizations, or those of the publisher, the editors and the reviewers. Any product that may be evaluated in this article, or claim that may be made by its manufacturer, is not guaranteed or endorsed by the publisher.
