# Supplementary material for: Supplementation of Kiwifruit Polyphenol Extract Attenuates High Fat Diet Induced Intestinal Barrier Damage and Inflammation via Reshaping Gut Microbiome
Source: Front Nutr. 2021 Aug 30;8:702157. doi: 10.3389/fnut.2021.702157 (PMC8435571; doi:10.3389/fnut.2021.702157)
Supplement: Supplementary file 1 [file Table_1.docx]

Supplementary results

The identification of polyphenols on kiwifruit peel extract:

The polyphenol composition of kiwifruit peel was used for HPLC-PDA analysis, and detecting conditions are as follows: the analytical column was the Diamonsil C18(250 mm×4.6 mm,5 µm, Agilent Technologies Inc., California, USA). Mobile phase A was100% methanol and mobile phase B was 0.5% acetic acid in water. And the solvent gradient was as follows: 0-10 min,10% A; 10-20 min, 13% A; 20-27 min, 18% A; 27-35 min, 22% A; 35-47min 25% A; 47-50 min 32%A; 50-58 min 35% A; 58-70min 10 A%. Elution was performed at a flow rate of 0.5 mL/min during 20 to 58min, and others time was 1.0 mL/min. And the injection volume was 10 μL and the column was thermostated at 35 °C. The analysis wavelengths were 280 nm for quantifying the polyphenols.

Table 1 The linear regression equation, correlation coefficient, linear range and detection limit of HPLC-PDA analysis on kiwifruit polyphenol extract

| Compounds | Linear equation | R^2^ | Linear range /(μg/mL) | detection limit /(mg/g) |
| --- | --- | --- | --- | --- |
| Gallic acid | y=9500x-59.482 | 0.9997 | 12.25~400 | 0.006 |
| Protocatechin | y=3006.7x+22.761 | 0.9994 | 12.25~400 | 0.002 |
| Chlorogenic acid | y=5710.1x-5.802 | 0.9997 | 12.25~400 | 0.005 |
| 6,7-Dihydroxycoumarin | y=2504.4x+6.893 | 0.9993 | 12.25~400 | 0.006 |
| Catechin | y=12485x-397.64 | 0.9992 | 25.00~400 | 0.004 |
| Epicatechin | y=1994.5x+36.575 | 0.9996 | 12.25~400 | 0.002 |
| P-coumaric acid | y=3769.5x+29.524 | 0.9998 | 12.25~400 | 0.007 |


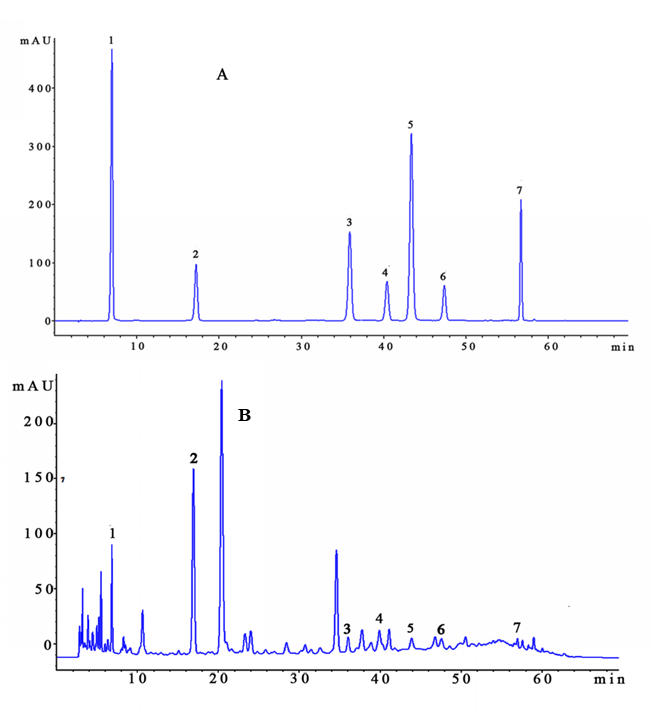
Figure 1: The HPLC-PDA chromatogram and UV-VIS scanning spectra of kiwifruit polyphenol extract: (A) HPLC-PDA chromatogram and UV-VIS scanning spectra of the standard polyphenols at 280 nm and peak identities (1: Gallic acid 2: Protocatechuic 3: Chlorogenic acid 4: 6,7-Dihydroxycoumarin 5: Catechin 6: Epicatechin 7: P-coumaric acid);(B) HPLC-PDA chromatogram and UV-VIS scanning spectra of the kiwifruit peel polyphenol extract at 280 nm and peak identities (1: Gallic acid 2: Protocatechuic 3: Chlorogenic acid 4: 6,7-Dihydroxycoumarin 5: Catechin 6: Epicatechin 7: P-coumaric acid);
